# CD8 immunoPET imaging to stratify response and guide combination immunotherapy and radiation in triple negative breast cancer

**DOI:** 10.1186/s13058-026-02286-9

**Published:** 2026-04-25

**Authors:** Patrick N. Song, Chloe T. DeMellier, Shannon E. Lynch, Carlos A. Gallegos, Ameer Mansur, Jonathan A. Moye, Alessandro Mascioni, Fang Jia, Christopher D. Willey, Anna G. Sorace

**Affiliations:** 1 Department of Radiology, The University of Alabama at Birmingham, Birmingham, USA; 2 Graduate Biomedical Sciences, The University of Alabama at Birmingham, Birmingham, USA; 3 Department of Biomedical Engineering, The University of Alabama at Birmingham, Birmingham, USA; 4 Imaginab, Inc, Inglewood, CA USA; 5 Department of Radiation Oncology, The University of Alabama at Birmingham, Birmingham, USA; 6 O’Neal Comprehensive Cancer Center, The University of Alabama at Birmingham, Birmingham, USA

**Keywords:** 4T1, Radiation resistance, TNBC, Df-IAB42

## Abstract

**Purpose:**

PET imaging targeting immune cells can be used to dynamically monitor intratumoral immune modulation in tissues. Radiation therapy is known to alter the tumor immune microenvironment; therefore, this study demonstrates how CD8 immunoPET imaging can optimize combination immunotherapy and radiation therapy by stratifying tumors who could derive the greatest benefit from immunotherapy following radiation therapy.

**Experimental design:**

A radiation resistant triple negative breast cancer cell line was derived through repeat irradiation of the radiosensitive parental 4T1 cell line prior to in vivo studies, until a radiation resistant subclone (RR-4T1) was isolated. CD8 immunoPET imaging was used to image immune cell infiltration in response to fractionated radiotherapy in radiation sensitive and radiation resistant 4T1 breast cancer models. In this genetically matched radiation sensitive and resistant model, we explore how radiation resistance alters radiation-induced immune modulation and CD8 T cell trafficking with flow cytometry, while response to combination radiation and immunotherapy was assessed in the radiosensitive parental 4T1 model. CD8 immunoPET was utilized to stratify for long-term therapeutic response to immunotherapy based on post-radiation therapy changes in CD8 tissue infiltration.

**Results:**

Radiosensitive parental 4T1 tumors show increased CD8 immunoPET signal (SUV) when treated with radiation therapy, relative to control tumors (*p* < 0.01) whereas radiation resistant 4T1 tumors showed no change following radiation therapy (*p* = 0.99), which was validated with flow cytometry. When tumors were stratified for high or low CD8 minibody uptake, CD8-high radiosensitive parental 4T1 tumors treated with radiation and immunotherapy had significantly increased sensitivity to immunotherapy compared to CD8-low radiosensitive parental 4T1 tumors (*p* < 0.05).

**Conclusion:**

Radiation therapy enhanced CD8 + expression in tumors and CD8 immunoPET effectively stratifies tumors that are more likely to respond to subsequent immunotherapy. CD8 immunoPET provides an approach to optimize combination immunotherapy following radiation treatment in triple-negative breast cancer.

**Supplementary Information:**

The online version contains supplementary material available at 10.1186/s13058-026-02286-9.

## Introduction

The tumor immune microenvironment (TIME) favors immunosuppression, which reduces a wide range of types of therapies efficacy [1–4]. Tumors have been observed to overexpress Programmed Death Ligand 1 (PD-L1) or Cytotoxic T-Lymphocyte Antigen 4 (CLTA-4) [5], which decreases the cytotoxic capacity of T cells, thereby reducing the impact of immunotherapy (IO) [5]. Standard-of-care for triple negative breast cancers (TNBC) incorporates combinations of chemotherapy, radiation, and IO; however, a subset of TNBC present with an immune-cold TIME, thereby decreasing IO efficacy [6, 7].

Radiation therapy (RT) is a key component of treatment for breast cancer care and management [8–10]. In stage I-III TNBC, RT is typically used in a post-surgical setting to prevent recurrence of micrometastases [11], and in late stage IV TNBC, RT is used to treat metastatic tumors that do not respond to systemic treatment [12]. High doses of RT (> 10 Gy) have been shown to induce modulation of the TIME resulting in increased innate and adaptive immune cell changes through increased expression of pro-inflammatory cytokines such as TNF-α, IFN-γ, IL-6 and IL-12 [13–19]. While RT is known to control tumor growth by inducing DNA double strand breaks (DSB), it has also been observed to promote an anti-tumoral immune response through increased CD8 T cell trafficking and increased polarization of M0 macrophages to M1 macrophages [1, 20–23]. As immunotherapies are known to be dependent on T cells, assessing changes in immune infiltration is critical for sequencing and timing of combination therapies [24, 25].

Immune profiling of tumors is typically characterized with immunohistochemistry [26–28]; however, immunohistochemistry represents a subsection of the tumor [29–31]. Immune positron emission tomography (i.e. immunoPET) imaging utilizes the noninvasive advantage of PET to allow for assessment of immune trafficking and has demonstrated a role in predicting therapy response to IO [32–37]. Wu et al. previously demonstrated that CD8 immunoPET imaging can detect changes in systemic and tumor associated CD8 T cells during therapy [38]. CD8 immunoPET imaging, using the Df-IAB42 CD8 targeted minibody, has also been used to understand the underlying immune infiltration in glioblastoma preclinical models following oncolytic herpes simplex viral and checkpoint blockade immunotherapy [34, 37]. PET imaging of immune populations have been utilized to study radiation-immunotherapy combinations, however most prior studies have focused on the systemic immune response or single timepoint assessments following the sequential combination therapy [38, 39]. As a result, there remains a limited understanding of how intratumoral immune dynamics change longitudinally during radiation therapy and how these changes relate to secondary therapeutic response to immunotherapy.

Immune infiltration, particularly stromal tumor-infiltrating lymphocytes (sTILs), is a well-established prognostic and predictive biomarker of response in TNBC. However, the majority of studies assessing sTILs have focused on pre-treatment tissue samples, which limits insights into how immune infiltration changes during therapy, particularly radiotherapy. Furthermore, repeated tissue sampling is invasive and therefore is typically completely only at the initiation of trials, thereby restricting the ability to longitudinally assess how treatments modulate the immune system. Consequently, there remains an unmet clinical need for noninvasive biomarkers that can longitudinally monitor how intratumoral immune responses change during treatments such as radiotherapy. If tumors can be modulated to have increased immune stimulation following radiation, it can allow for potential sensitization for secondary immunotherapies. This imaging approach provides an opportunity to identify tumors most likely to benefit from combination sequential radiation-immunotherapy strategies. This study characterizes how CD8 immunoPET imaging can monitor modulation in CD8 T cells in response to RT across radiosensitive and radioresistant syngeneic TNBC models and evaluates whether early post-radiotherapy CD8 dynamics can stratify tumors for response to subsequent IO. This study addresses a gap in research by identifying which tumors benefit most from RT + IO and paves the way for the development of personalized treatments in TNBC. By understanding the dynamics of CD8 immune infiltration following cytotoxic therapy, there is potential to improve therapeutic outcomes by optimizing timing and combination therapy of TNBC.

## Materials and methods

### Syngeneic model of radiation sensitive and resistant breast cancer

**Fig. 1:**
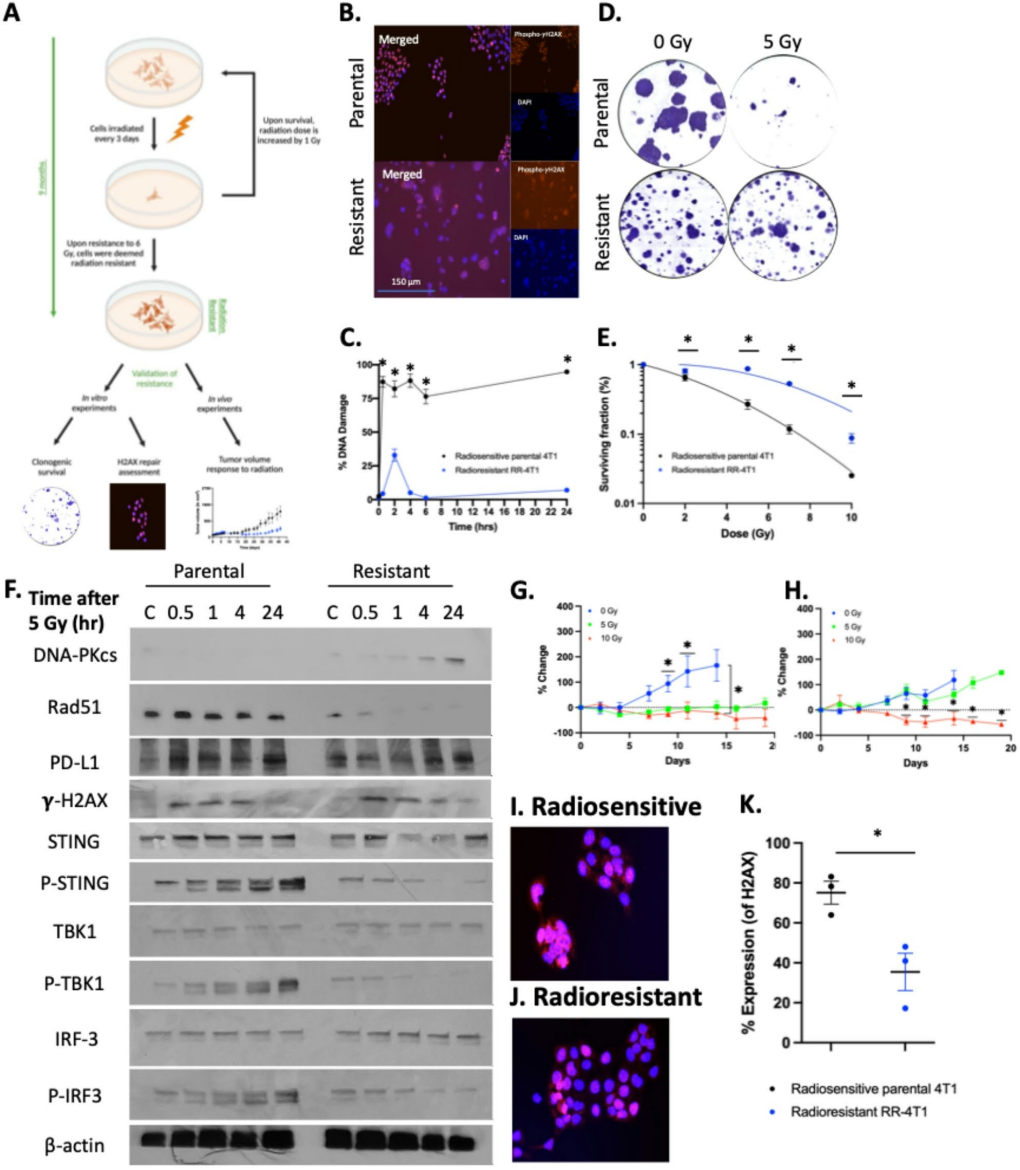
Molecular profiling of radiation resistance in 4T1 cells reveals significant alterations in survival and DNA damage repair. Schema for acquired radiation resistant in syngeneic models of TNBC (**A**). Representative immunofluorescence staining of γH2AX in radiosensitive parental and radioresistant RR-4T1 cells following a single 12 Gy RT dose (**B**). Quantification of γH2AX foci intensity from T = 0 to 24 h post RT in radiosensitive parental 4T1 and radioresistant RR-4T1 cells (**C**). Representative clonogenic survival assays and quantification of survival performed in radiosensitive parental 4T1 and radioresistant RR-4T1 cells (**D**–**E**). Western blotting of DNA-PKcs, Rad51, STING, p-STING, TBK1 p-TBK1, IRF-3, and p-IRF-3 in radiosensitive parental 4T1 and radioresistant RR-4T1 cell lines in response to RT (**F**). In vivo tumor growth characterization following single dose 5–10 Gy radiation in radiosensitive parental 4T1 (**G**) or radioresistant RR-4T1 tumors (**H**). γH2AX immunofluorescence staining of radiosensitive parental 4T1 (**I**) and radioresistant RR-4T1 tumors in vivo following 5 Gy radiotherapy (**J**) with quantification (**K**)

4T1 and EMT6 cells were acquired and authenticated from ATCC and were maintained in RPMI 1640 (Fisher Scientific Catalog #: 11875093) supplemented with 10% fetal bovine serum. To develop acquired radioresistance, radiosensitive parental 4T1 cells were cultured in tissue culture flasks in vitro and irradiated with 2 Gy using an XRAD320 irradiator (Precision X-Ray Inc., N. Bradford, CT, USA) every 3–4 days while maintained under standard growth conditions. Irradiation was performed directly on adherent cells in flasks and, between irradiation, cells were allowed to recover and proliferation. When cells reached 70–80% confluence, the irradiation dose was increased by 1 Gy (until 6 Gy). The time required to establish resistance at each radiation dose varied, with resistance developing more rapidly at lower doses and requiring longer adaptation periods at higher doses. Once resistance to 6 Gy was established, cells were irradiated weekly with 6 Gy to maintain resistance (Fig. 1A). Surviving cells were referred to as radiation resistant 4T1 cells or “RR-4T1” and all in vivo and imaging experiments were performed within a year of resistance establishment. Six-week-old female BALB/c mice were obtained from Charles River Laboratories (catalog number: 028) and orthotopically injected into the mammary fat pad with radiosensitive parental 4T1 or radioresistant RR-4T1 cells under general anesthesia using 2% isofluorane in oxygen. At experimental baseline, all tumors were between 100 and 150 mm^3^. To assess in vivo radioresistance, radioresistant RR-4T1 tumors were irradiated with 0, 5, or 10 Gy and measured thrice weekly to determine tumor viability (*N* = 4–6). RT was delivered under general anesthesia with 2% isoflurane in oxygen. Tumor-only irradiation was achieved using lead shielding to protect non-target tissue. An additional cohort of radiosensitive parental 4T1 and radioresistant RR-4T1 tumors were engrafted and assessed for γH2AX expression. Radiosensitive parental 4T1 and radioresistant RR-4T1 tumors were irradiated with 5 Gy RT and, 24 h later, excised for immunofluorescence staining with 1:100 anti-phospho-$\:\gamma\:$ H2AX (Cell Signaling Catalog #: 803125) (*N* = 3). Tumors were formalin fixed for 48 h prior to paraffin embedding. Paraffin embedded tumor sections were processed in accordance with published literature [10, 40]. Briefly, 5 μm slices were incubated with EZ-DeWax (BioGenex. Catalog #: HK584-5 K) for approximately 10 min at room temperature and subsequently incubated with Antigen Retrieval Buffer (Abcam Catalog #: ab93678) for 10 min at 100° C. Slides are then blocked with 5% BSA in TBS-T for 30 min at room temperature and incubated with primary anti-phospho-$\:\gamma\:$ H2AX antibody overnight at 5° C. Following this, slides are incubated with 1:100 Alexa Fluor 647 AffiniPure Donkey Anti-Mouse IgG (Thermofisher Scientific Catalog #715-605-150) for one hour. Tumor sections are then washed, counterstained with DAPI and visualized with an EVOS M7000 imaging system (Thermofisher Scientific, Waltham, MA, USA) with DAPI (Excitation/emission: 357–444/447–460 nm) and Cy5 (Excitation/emission: 628–640/692–740 nm) settings. The percentage of γH2AX cells was calculated using custom MATLAB scripts that identified and quantified DAPI+ nuclei and DAPI/Cy5 double-positive cells. For each tumor, four randomly selected 20x fields of view (513 × 382 microns) within viable tumor regions were analyzed. Cells were segmented using DAPI-stained nuclei and a γH2AX-positive cell was defined as a nucleus containing ≥ 20 discrete γH2AX foci. The percentage of γH2AX-positive cells was calculated as the number of γH2AX-positive cells divided by the total number of DAPI-positive nuclei within each field of view. Values from four fields were averaged to generate a single value per tumor. Image processing, segmentation, and quantification were performed using custom MATLAB scripts (Supplementary File 1), which included automated identification of nuclei, detection of γH2AX foci, and application of predefined thresholds for positivity.

*Molecular probing of acquired radiation resistance in 4T1 cells* in vitro.

To assess biomarkers of DNA repair, radiosensitive parental 4T1 and radioresistant RR-4T1 cells were treated with 5 Gy RT. At timepoints 0.5, 1-, 4-, and 24-hours post RT, cells were lysed for western blot. Lysate was run through a Mini-Protean TGX Precast Gel (BioRad Catalog #: 4561096) and transferred with a Trans-Blot Turbo transfer pack (BioRad Catalog #: 1704157). The membrane was probed for Rad51 (Abcam Catalog #: ab88572), DNA-PKcs (Thermofisher Scientific Catalog #: MA5-32192), PD-L1 (Thermofisher Scientific Catalog #: PA5-20343), anti-phospho-$\:\gamma\:$ H2AX (Cell Signaling Catalog #: 803125), anti-STING (Cell Signaling Catalog #: 13647 S), anti-phospho-STING (Thermofisher Scientific Catalog #: PA5-105674), anti-TBK1 (Cell Signaling Catalog #: 3013 S), anti-phospho-TBK1 (Cell Signaling Catalog #: 5483 S), anti-IRF3 (Cell Signaling Catalog #: 4302 S), anti-phospho-IRF3 (Cell Signaling Catalog #: 4947 S), and β-actin (Abcam Catalog #: ab49900). Following incubation, the membrane was stained with HRP anti-rabbit IgG (Cell Signaling Technology Catalog #: 7074 S) or HRP anti-mouse IgG (Cell Signaling Technology Catalog #: 7076 S) antibody, developed with Pierce ECL Western Blot Substrate (Fisher Scientific Catalog #: 32209) and visualized with CL-XPosure Film (Fisher Scientific Catalog #: 34089).

*Characterizing changes in DNA damage response and clonogenic survival in radiation resistant TNBC* in vitro.

Immunostaining was conducted to assess DNA damage between radiosensitive parental and radioresistant RR-4T1 cells. Parental (*N* = 8) and RR (*N* = 3–8) cells were plated and treated with 12 Gy RT. Cells were fixed at 0, 2-, 4-, 6-, and 24-hours following treatment. Cells were stained with 1:250 anti-phospho-$\:\gamma\:$ H2AX (Cell Signaling Catalog #: 803125) and, following incubation, were stained with 1:100 Alexa Fluor 647 AffiniPure Donkey Anti-Mouse IgG (Thermofisher Scientific Catalog #715-605-150). Cells were imaged using the EVOS M7000 with DAPI (Excitation/emission: 357–444/447–460 nm) and RFP (Excitation/emission: 531–540/593–640 nm) channels. Total DNA damage was quantified by assessing co-localization of cells expressing phospho-$\:\gamma\:$ H2AX and DAPI (Supplemental File 1). Clonogenic survival of radiosensitive parental and radioresistant RR-4T1 cells in response to irradiation (0, 2, 5, 7, 10, or 20 Gy) was assessed with a clonogenic assay. Immediately following irradiation, radiosensitive parental and radioresistant RR-4T1 cells were plated with a seeding density of 100 and 1,000 cells/well respectively and then incubated undisturbed for 7 days (*N* = 3). Following incubation, plates were fixed and stained with 0.01% crystal violet (Fisher Scientific Catalog #AC40583025, Waltham, MA, USA). To determine plating efficiency, radiosensitive parental 4T1 and radioresistant RR-4T1 cells were seeded onto a 12-well plate at seeding densities of 100, 250, 500, and 1000 cells/well. Cells were incubated and allowed to grow undisturbed for 7 days (*N* = 3). Data was fitted using a linear-quadratic model, as shown in Fig. 1E (α and β parameters included in Supplementary Table 1).

### CD8 immunoPET radiochemistry

For all CD8 immunoPET experiments, the CD8 targeted minibody, Df-IAB42, was radiolabeled with [^89^Zr] according to previous studies [37, 40]. Briefly, [^89^Zr] was buffered with HEPES and adjusted to approximately 7.0 pH using sodium hydroxide and hydrochloric acid. Following pH adjustment, [^89^Zr] was incubated with 370 kBq/µg (10 µCi/µg) Df-IAB42 at 500 RPM for one hour at 37° C using a Thermomixer. Following this, purity was assessed using instant thin layer chromatography (iTLC) with 50 mM diethylenetriamine pentaacetate (DTPA). Animal imaging experiments were only conducted if [^89^Zr]Zr-Df-IAB42 radiolabeling efficiency exceeded 95% successfully labeled.

### CD8 immunoPET imaging to assess immune infiltration following RT

To determine if immune infiltration is predictive of response to RT, radiosensitive EMT6, radiosensitive parental 4T1 and radioresistant RR-4T1 tumors were irradiated and imaged with CD8 immunoPET. Mice were randomized into treatment groups once tumors reached 100–150 mm^3^ to ensure comparable baseline tumor growth across groups. Upon enrollment into the study, tumors were treated with 2 Gy x 6 fractionated RT on days 0–5. On day 6, mice were injected with 1.85MBq (50 µCi) of [^89^Zr]Zr-Df-IAB42, a minibody with specificity for CD8, and imaged 24 h later with a small animal PET/CT (Sofie Biosciences, Somerset, NJ, USA). During PET/CT, mice were anesthetized with 2% isoflurane anesthesia in oxygen. Static PET imaging was acquired for 20 min using all PET beds, which were randomized across experimental groups. PET/CT images were processed and analyzed using VivoQuant. Standardized uptake values (SUV) were calculated as activity concentration normalized to injected dose and body weight. Frequency analysis was conducted to assess the voxel-wise distribution of intratumoral SUV values, reflecting changes in overall heterogeneity in CD8-associated PET signaling. Following imaging, a subset of mice was humanely euthanized by isoflurane overdose followed by cervical dislocation for biological validation, while remaining mice were monitored for tumor group for up to four weeks. For radiosensitive parental 4T1 tumors, control (*N* = 5) and radiation-treated (*N* = 10) groups were included. For radiation resistant RR-4T1 tumors, control (*N* = 5) and radiation-treated (*N* = 9) groups were included. For radiosensitive EMT6 tumors, control (*N* = 4) and radiation-treated (*N* = 8) groups were included. In total, 41 mice were used for this experiment.

### Immunofluorescence validation of CD8 immunoPET

Following CD8 immunoPET imaging, tumors were stained for CD8 (BD Biosciences Catalog #: 550281), followed by Cy5-Goat anti-rat secondary antibody (Jackson Laboratories, Catalog #: 112-175-143). Sections were imaged with the EVOS M7000 with the DAPI and Cy5 settings. CD8 staining was quantified by normalizing Cy5-positive signal to DAPI-stained nuclei across four randomly selected 20x fields (513 × 382 microns) of viable tumor tissue. The percentage of CD8 + cells was calculated using custom MATLAB scripts that identified and quantified DAPI+ nuclei and DAPI/Cy5 double-positive cells.

### Identification of immune populations in radiation sensitive and radiation resistant TNBC models

To identify the immune populations driven by RT, radiosensitive parental 4T1 or radioresistant RR-4T1 tumors were grown to 100–150 mm^3^ and treated identically to CD8 immunoPET experiments. Tumors were mechanically digested with 1:100 type IV collagenase (Sigma Aldrich Catalog #: C5138) for 30 min at 37° C. Following digestion, tumors were washed with 1x PBS + 2% FBS and lysed in ACK lysis buffer (Thermofisher Scientific Catalog #: A1049201). Cells were then stained with eFluor450 (Thermofisher Scientific Catalog #: 65-0863-14) and subsequently incubated with FITC-CD4 (Thermofisher Scientific Catalog #: 11-0041-81), PerCP-Cy5.5-CD8a (Thermofisher Scientific Catalog #: 45-0081-80), APC-CD3 (Thermofisher Scientific Catalog #: 17-0031-63), AF700-IFNγ (Thermofisher Scientific Catalog #: 56-7311-80), BV421-CD80 (Biolegend Catalog #: 104725), BV510-CD45 (Thermofisher Scientific Catalog #: 563891), SB600-CD69 (eBioscience Catalog #: 63-0699-42), BV650-CD279 (BD Biosciences Catalog #: 564324), SB702-CD86 (Thermofisher Scientific Catalog #: 67-0862-82), BV785-MHCII (BD Biosciences Catalog #: 564324), PE-CD25 (Invitrogen Catalog #: 12-0251-83), and PE-Cy7-F4/80 (Thermofisher Scientific Catalog #: 25-4801-82) for 30 min at 5° C at manufacturer-recommended concentrations. Cells were then suspended in Fluorescence Activated Cell Sorting (FACS) buffer prior to being analyzed with flow cytometry. Cell populations were acquired with an Attune NxT Flow Cytometer (Thermofisher Scientific. Waltham, MA) and quantified with FlowJo 10.6.2 (TreeStar Inc, Ashland, OR). Immune cell populations were identified using a gating strategy that gated for singlets → live cells → CD45 + leukocytes, followed by immune cell specific markers to define T cells (CD3, CD4, and CD8), myeloid cells (F4/80, CD80, CD86, and MHCII) and activation markers (CD69, CD279, and IFN-γ). For radiosensitive parental 4T1 tumors, *N* = 4 mice were included in control groups and *N* = 10 in radiation-treated groups. For radiation resistant RR-4T1 tumors, *N* = 5 mice were included in control groups and *N* = 6 in radiation-treated groups, for a total of 25 mice in this analysis.

### CD8 immunoPET imaging to stratify response to IO by radiation driven immune modulation

To understand how RT driven immune infiltration affects response to IO, radiosensitive parental 4T1 tumors were imaged with CD8 immunoPET imaging following RT and treated with IO. Mice received an identical RT and CD8 immunoPET imaging regimen to prior experiments. Mice were administered with combination anti-PD1 (10 mg/kg) and anti-CTLA4 (5 mg/kg) every 3 days from day 9 until day 24. Mice treated with only IO began treatment on day 0, were treated every 3 days (until day 18), and were imaged on day 7. K-means thresholding was utilized to stratify tumors with low CD8 immune populations or high CD8 immune populations after RT. A response threshold was set at 30% increased tumor volume relative to the tumor volume on day 7. Experimental group sizes included *N* = 8 control, *N* = 3 IO-only, *N* = 13 RT-only, and *N* = 15 RT → IO mice, for a total of 39 mice in this analysis. The immunotherapy only cohort was included to provide contextual reference for lack of response to combination treatment response, and demonstrate consistency to that shown repeatedly in previous literature [41, 42], rather than a central experimental arm.

### Statistical analysis

Experimental conditions were summarized by average of replicates and error was represented by standard error of mean (SEM). Due to the nature of the experimental design, treatments were not blinded. Significance between experimental conditions was assessed using a Mann-Whitney U test. For tumor growth studies, statistical comparisons were performed at the final experimental timepoint. Flow cytometry analyses of individual immune cell populations were performed as independent comparisons. All data and figures were analyzed and generated using GraphPad Prism 7 (La Jolla, CA, USA). A p-value < 0.05 was considered statistically significant.

## Results

### Repeated irradiation generates a syngeneic radiation resistant model of TNBC with altered DNA damage repair and survival

To monitor changes in DNA damage response, radiosensitive parental 4T1 and radioresistant RR-4T1 cells were assessed for DNA damage (Fig. 1B). Radioresistant RR-4T1 cells consistently showed decreased γH2AX expression, relative to radiosensitive parental 4T1 cells (*p* < 0.01) (Fig. 1C). To assess clonogenic survival of radioresistant RR-4T1 cells, a clonogenic assay was conducted and radioresistant RR-4T1 cells demonstrate increased survival at all doses of RT except 20 Gy RT (Fig. 1D and E and Supplemental Fig. 1). Clonogenic survival data were additionally fit using the linear-quadratic model to derive α and β parameters and α/β ratio for radiosensitive parental 4T1 and radioresistant RR-4T1 cells (Supplemental Table 1). To assess changes in molecular expression in radioresistant RR-4T1 cells, a western blot was performed on radiosensitive parental 4T1 and radioresistant RR-4T1 cells after exposure to 5 Gy radiotherapy (Fig. 1F). Western blot analysis reveals differential expression of DNA damage repair proteins (Rad51, and DNA-PKcs) between radiosensitive parental 4T1 and radioresistant RR-4T1 cells. While these findings do not directly measure HR/NHEJ activity, they provide correlative evidence for distinct repair pathways between these two models. Further western blot analysis revealed increased expression of pSTING, pTBK1 and pIRF3 in radiosensitive parental 4T1 cell line when treated with RT; however, no increase in pSTING, pTBK1 and pIRF3 was observed in radioresistant RR-4T1 cells in response to RT. In vivo, radiosensitive parental 4T1 tumors demonstrated a reduction in tumor volume in response to 5 Gy and 10 Gy RT (*p* = 0.057, *p* = 0.056; Fig. 1G), while radioresistant RR-4T1 tumors demonstrated no response at 5 Gy (*p* = 0.25), but a significant response to 10 Gy RT (*p* < 0.01; Fig. 1H). Following RT (5 Gy, 24 h post-RT), radiosensitive parental 4T1 and radioresistant RR-4T1 tumors were assessed for γH2AX and representative immunofluorescence images demonstrate qualitatively higher γH2AX signaling in radiosensitive parental 4T1 tumors when compared to radioresistant RR-4T1 tumors (Fig. 1I and J). Radiosensitive parental 4T1 tumors, when treated with 5 Gy RT, compared to radioresistant RR-4T1 tumors (*p* = 0.022; Fig. 1K). With the increased clonogenic survival in vitro and reduced response in vivo to 5 Gy RT, these findings support the classification of RR-4T1 as a radiation resistant TNBC model.

### CD8 immunoPET imaging monitors immune infiltration and heterogeneity following fractionated RT in TNBC

**Fig. 2:**
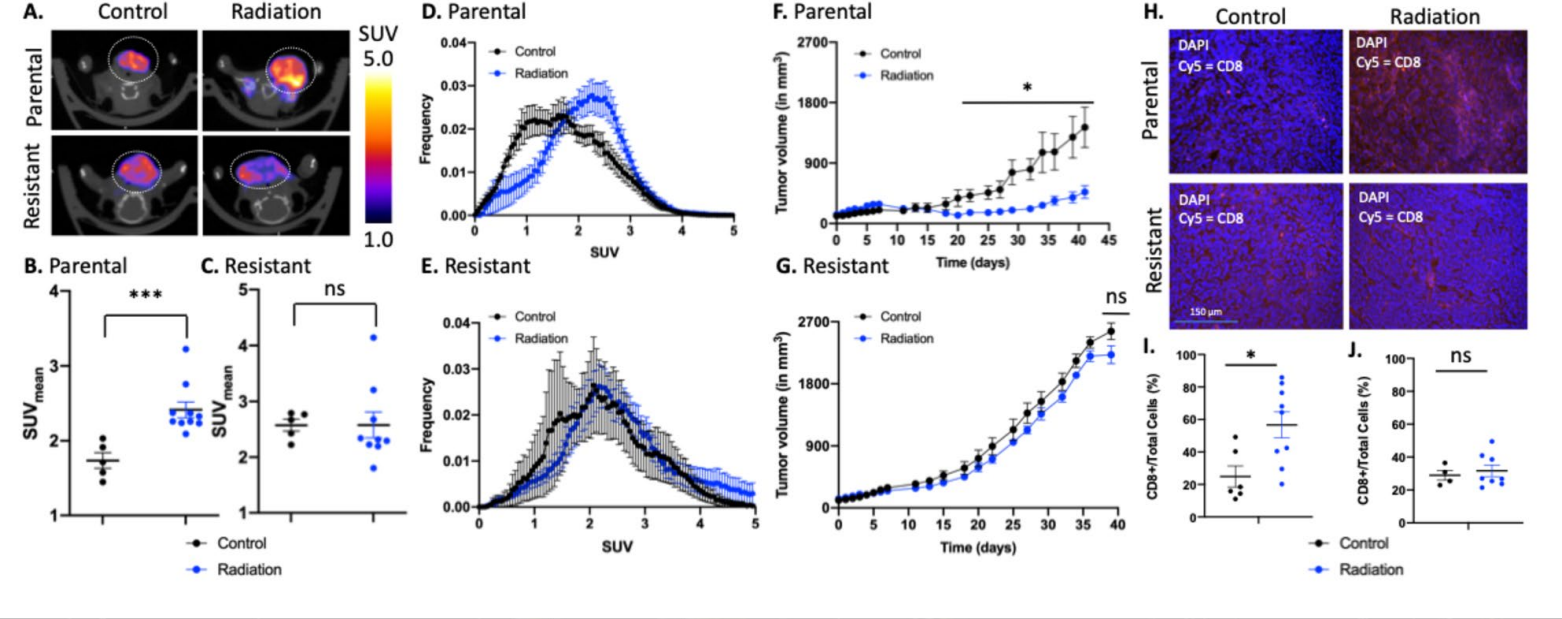
CD8 immunoPET reveals increases in CD8 immune infiltration in response to RT in radiosensitive parental 4T1 tumors. Representative CD8 immunoPET imaging of radiosensitive parental 4T1 and radioresistant RR-4T1 tumors under control and irradiated conditions (**A**). Quantification of intratumoral CD8 SUV in radiosensitive parental 4T1 (**B**) and radioresistant RR-4T1 tumors (**C**). Frequency distribution of voxel-wise CD8 SUV values in radiosensitive parental 4T1 (**D**) and radioresistant RR-4T1 tumors (**E**) following radiotherapy. Longitudinal tumor volume assessment for radiosensitive parental 4T1 (**F**) and radioresistant RR-4T1 tumors (**G**) following control or fractionated RT. Representative immunofluorescence images of tumor sections stained for CD8 (**H**). Quantification of CD8 immunofluorescence staining in radiosensitive parental 4T1 (**I**) and radioresistant RR-4T1 tumors (**J**) following control or fractionated RT

To characterize immune infiltration following RT, [^89^Zr]Zr-Df-IAB42 PET imaging allowed for quantification of CD8 infiltration in parental and radiation resistant TNBC models (Fig. 2A). In radiosensitive parental 4T1 tumors, irradiated tumors had significantly increased CD8 SUV compared to controls (*p* < 0.01) (Fig. 2B), which was validated in a second syngeneic radiosensitive TNBC cell line EMT6 (*p* < 0.01) (Supplemental Fig. 2) [43]. Conversely, in radioresistant RR-4T1 tumors, no significant change in imaging metrics was observed between controls and RT treated tumors (*p* = 0.99) (Fig. 2C). Frequency analysis of radiosensitive parental 4T1 and radioresistant RR-4T1 tumors shows that RT skews the intratumoral distribution of CD8-PET signal toward higher SUV values in parental tumors (Fig. 2D) but not in radiation resistant tumors (Fig. 2E). In radiosensitive parental 4T1 tumors, a significant decrease in tumor viability is observed on day 20 in response to RT, which is sustained longitudinally (Fig. 2F). This decrease in tumor volume is not observed in RR-4T1 tumors (Fig. 2G). CD8-PET imaging shows increased CD8 signal suggestive of higher CD8 + infiltration, and this interpretation was supported by immunofluorescence staining demonstrating increased CD8 immunostaining in parental tumors after RT and no significant change in RR-4T1 tumors (Fig. 2H-J). The correlation between CD8 immunoPET SUV and CD8 immunofluorescence staining reveals a moderate significant correlation (R^2^ = 0.27, *p* < 0.01) (Supplemental Fig. 3). These findings support that changes in CD8 immunoPET SUV reflect differences in CD8 T cell infiltration following RT in radiosensitive 4T1 tumors.

### RT drives an increased inflammatory response in radiation sensitive TNBC

**Fig. 3:**
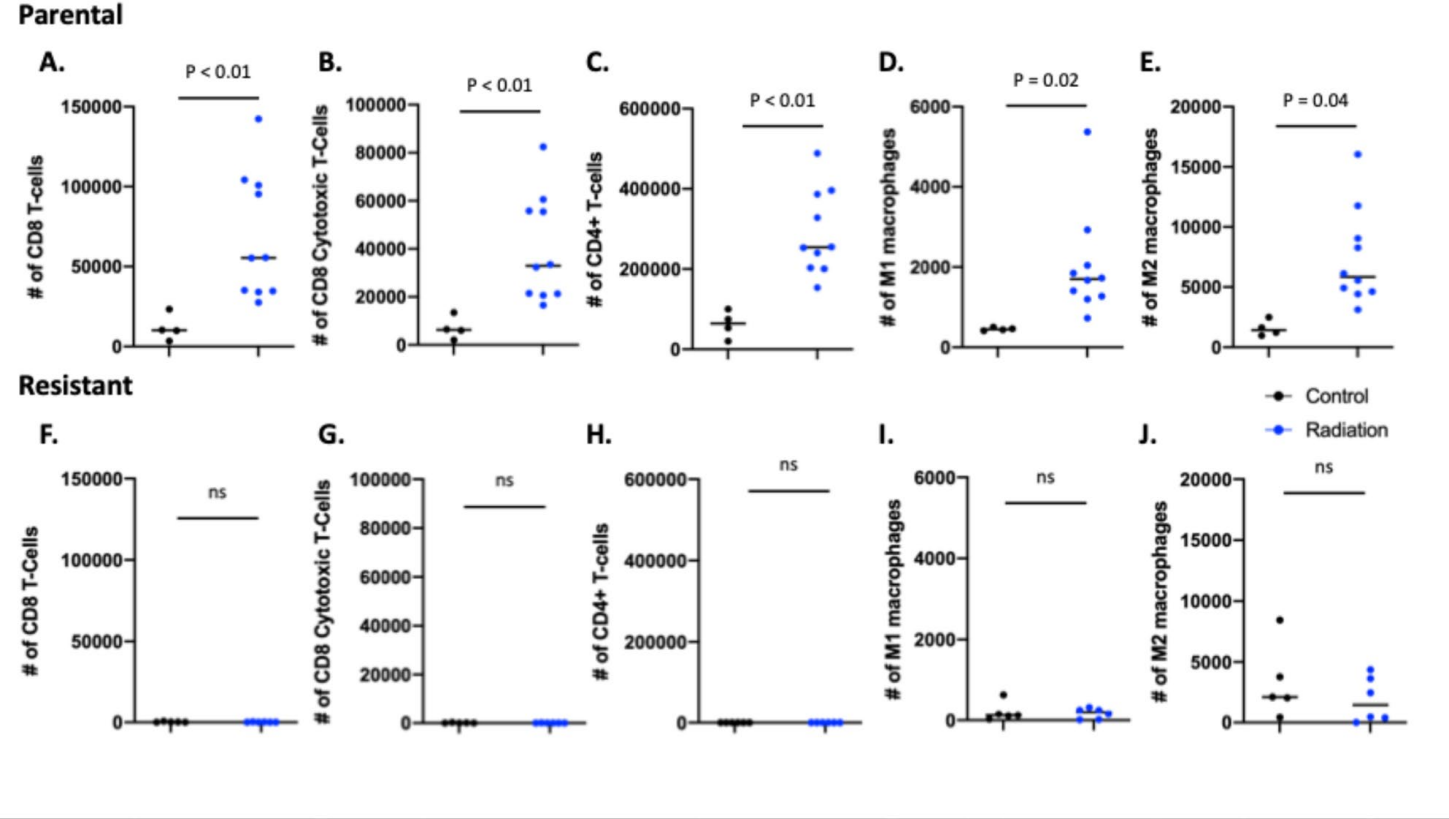
RT induces an inflamed TIME in radiation responsive tumors. Radiosensitive parental 4T1 and radioresistant RR-4T1 tumors were treated with RT and analyzed with flow cytometry for changes in immune cell populations. Absolute numbers of CD8 + T cells (**A**, **F**), cytotoxic CD8 + T cells (**B**, **G**), CD4 + T cells (**C**, **H**), M1-like macrophages (**D**, **I**) and M2-like macrophages (**E**, **J**) are shown for radiosensitive parental 4T1 tumors (A-E) and radioresistant 4T1 tumors (F-J)

To identify immune changes following RT, tumors were treated with RT and probed for immune populations with flow cytometry. At baseline, radiation sensitive parental 4T1 tumors demonstrate increased CD8, cytotoxic IFN-γ CD8 + T cells and CD4 + T cells compared to radioresistant RR-4T1 tumors (Supplemental Fig. 4). In radiosensitive parental 4T1 tumors, RT led to a significant increase in CD8 + T cells (Fig. 3A) and cytotoxic IFN-γ CD8 + T cells (Fig. 3B) when compared to controls (*p* < 0.01). RT significantly increased the number of CD4 + T cells, relative to controls (*p* = 0.001) (Fig. 3C). In radiosensitive parental 4T1 tumors, RT significantly increased the number of M1-like and M2-like macrophages, relative to those in tumors treated with control (*p* = 0.015) (Fig. 3D-E). Conversely, in radioresistant RR-4T1 tumors, no significant difference in immune infiltration was observed after irradiation (Fig. 3F–J). In addition, RR-4T1 tumors exhibit lower overall immune cell abundance compared with parental 4T1 tumors, consistent with a more immune-excluded TIME associated with acquired radiation resistance.

### CD8 immunoPET imaging thresholds CD8 immune infiltration and informs on response to IO

**Fig. 4:**
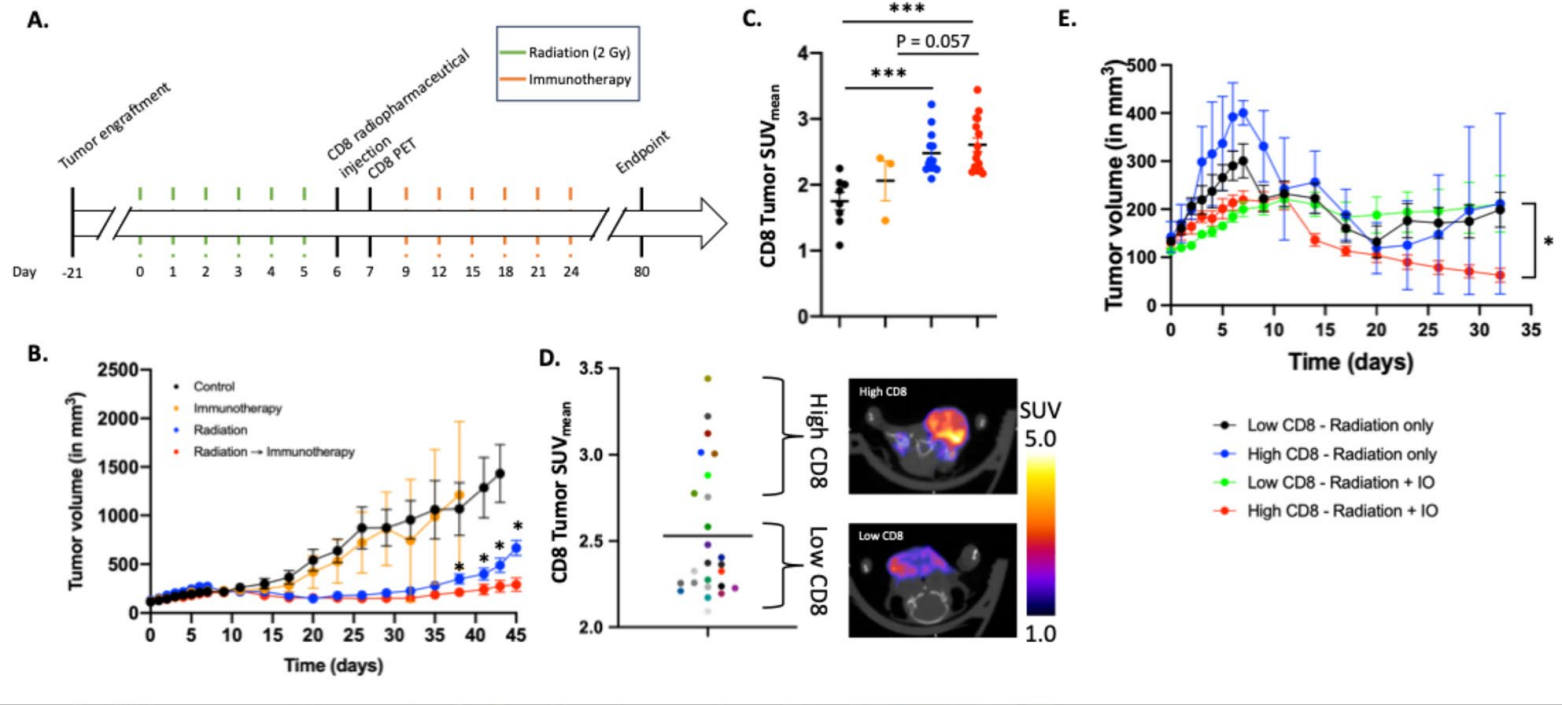
CD8 immunoPET imaging successfully thresholds response to IO in preclinical radiation responsive TNBC. Experimental timeline to assess the utility of CD8 immunoPET imaging to identify post-radiation immune infiltration and stratify response to immunotherapy (**A**). Longitudinal tumor volume assessment of radiosensitive parental 4T1 tumors treated with control, IO-only, RT-only and RT → IO (**B**). Quantification of intratumoral CD8 immunoPET SUV in radiosensitive parental 4T1 tumors treated with control, IO-only, RT-only and RT → IO (**C**). CD8 immunoPET SUV stratification of irradiated tumors into high CD8 and low CD8 groups through K-means clustering (**D**). Segmenting changes in RT and RT → IO response with CD8 immunoPET imaging (**E**)

To assess the efficacy of CD8 immunoPET imaging to guide IO following RT, tumors were monitored for intratumoral CD8 populations following RT and prior to subsequent IO (Fig. 4A). Radiosensitive parental 4T1 tumor bearing mice treated with IO had a similar response to controls (Fig. 4B). Conversely, RT and RT → IO treated tumors showed significant reductions in tumor volume (*p* < 0.01, Fig. 4B). RT → IO treated tumors began to show significant decreases compared to RT only treated tumors beginning on day 38 (*p* = 0.03). Tumors treated with RT and RT → IO consistently show increased CD8 SUV (*p* < 0.01). While CD8 SUV values were higher in RT → IO treated tumors compared to IO alone, this difference did not reach statistical significance (*p* = 0.057) (Fig. 4C). RT → IO treated tumors was separated into “High CD8” and “Low CD8” categories, stratified with K-means clustering of CD8 SUVmean values measured on day 7, and “high CD8” groups had 100% response to IO compared to “low CD8” groups which had 33.3% response (Fig. 4D, Supplemental Fig. 5). Due to experimental scope, combination radiation-IO response was evaluated exclusively in the radiosensitive parental 4T1 model and was not assessed in the radiation resistant RR-4T1 model. Further analysis of post-RT CD8 immunoPET SUV identified that CD8-based stratification was predictive of response to combination RT → IO, but not to RT alone. Tumors with higher post-RT CD8 immunoPET SUV exhibited delayed tumor growth after RT → IO, whereas tumors with lower CD8 immunoPET SUV showed a more heterogeneous response. In contrast, stratification of tumors by CD8 SUV did not reveal differences in tumor growth following RT alone; although a significant difference was observed between “Low CD8” and “High CD8” tumors treated with a combination of RT → IO (Fig. 4E). When CD8 SUV_mean_ was analyzed as a continuous predictor of response to RT → IO, linear regression revealed a weak non-significant association (R^2^ = 0.21, *p* = 0.10), indicating limited predictive performance in this cohort (Supplemental Fig. 6).

## Discussion

In this work, it is demonstrated that radiation therapy modulates CD8 in tumors and CD8 immunoPET can be used to effectively stratify tumors that are more likely to respond to subsequent immunotherapy. CD8 immunoPET imaging has been shown to be effective in predicting baseline CD8 expression in tumors [37, 40]. Two models of TNBC (4T1, EMT6) demonstrated the ability for CD8 PET to identify intratumoral differences in CD8 and a radiation resistant variant, RR-4T1, was developed to enhance the understanding of the relationship of radiation modulation and CD8 expression in tissue. This work adds to the growing body of literature that demonstrates how RT can alter tumor immunomodulation [21, 44–46] as radiation can drive increased immune infiltration through enhanced expression of DAMPs [21]. Preclinical studies in melanoma and colorectal cancers have similarly demonstrated a significant increase in CD8 effector T cell infiltration after radiation, however these studies focus on high dose irradiation, which is uncommon in clinical breast cancer treatment and has potential for higher toxicities [47, 48]. Combining RT and immunoPET has the potential to dynamically identify states of enhanced susceptibility to therapies that could benefit from enhanced immune infiltration [17, 49–52]. In a TNBC parental 4T1 model that is immunotherapy-resistant, CD8 immunoPET imaging was able to noninvasively identify immune infiltration following RT and stratify tumors that effectively respond to subsequent IO. As there is a wide range of response in clinical TNBC, quantifying the variability among tumor response and individually assessing biological changes when defining initial and updated personalized treatment plans could provide important information for clinical decision making.

The RR-4T1 model was developed to test the relationship of CD8 expression post radiation therapy to further our understanding of biological interactions in the tumor. The observed reduction in γH2AX foci intensity in radioresistant RR-4T1 cells suggests a possible mechanistic explanation for the impaired CD8 + T cell infiltration observed via CD8 immunoPET imaging. This reduction may reflect decreased radiation-induced DNA damage, which could result in attenuated DAMP signaling and impaired activation of the cGAS/STING pathway. A marked increase in the STING pathway was observed in radiosensitive parental 4T1 cells in vitro in response to RT, which was contrasted by expression in the radioresistant RR-4T1 cells. Attenuated cGAS-STING activation is known to reduce DNA damage per unit radiation dose and is expected to contribute to the diminished CD8 + T cell infiltration observed in radioresistant RR-4T1 tumors. In this study, radiation resistance was defined as reduced post-RT DNA damage response and decreased tumor growth inhibition relative to radiosensitive parental TNBC tumors at clinically relevant RT doses, rather than complete resistance at ablative doses. This single high-dose (12 Gy) radiotherapy exposure was intentionally used in vitro to assess acute DNA damage and repair mechanisms between the radiation sensitive and radiation resistant cell lines. For in vivo studies, 5 Gy and 10 Gy doses were delivered in clinically relevant fractions (2 Gy per fraction) and were utilized to evaluate tumor response and immune modulation. Notably, prior studies have demonstrated that 2 Gy fractionated RT can also induce immune modulation and enhance antitumor immune responses, including increased CD8 T cell infiltration and improved synergy with IO, highlighting dose per fraction and fractionation schedule as important variables influencing immunologic outcomes and warranting further investigation [13, 53]. Future studies integrating the immunocompetent in vivo models with expanded analysis of DNA damage repair pathways will be important to further define mechanisms of acquired radiation resistance. We observed a decrease in Rad51 in radioresistant RR-4T1 cell line in response to RT; however future studies into DNA damage repair pathways should include a traffic light reporter to better assess the temporal kinetics of DNA damage repair. In addition, the development of the radioresistant RR-4T1 variant, through iterative irradiation and validation, enabled us to address heterogeneity in radiation response, a key clinical challenge. However, baseline tumor growth kinetics differed between models, with radioresistant RR-4T1 cells exhibiting longer doubling rates as the cells showed increased resistance to RT (Supplemental Fig. 7). This reduced doubling rate indicates that impaired radiation induced immune modulation and diminished CD8 T cell trafficking observed in radioresistant RR-4T1 tumors are not attributed to increased tumor growth. Rather, this reflects biological adaptations acquired during radiation resistance. While the radioresistant RR-4T1 model demonstrates impaired radiation-induced modulation of CD8 T cells and reduced CD8 trafficking, evaluation of whether these features translate into resistance to combination radiation-IO should be studied in future directions.

ImmunoPET imaging using [^89^Zr]Zr-Df-IAB42, a CD8-targeted radiolabeled minibody, provides a noninvasive approach to quantifying immune dynamics of CD8 with corresponding downstream individual tumor response. Although CD8 immunoPET imaging specifically visualizes CD8 T cells, changes in CD8-associated signaling reflects the cumulative result of multiple immune-related processes including increased cytokine signaling and immune cell interactions. Identifying predictive biomarkers of response and characterizing the underlying tumor biology related to cancer growth and treatment response is critical in IO, as response rates are often heterogeneous. In this study, CD8 immunoPET imaging was used to characterize post-radiation immune heterogeneity and to stratify tumors based on their likelihood of responding to subsequent IO. This work further builds upon previous work studying the immune interaction of RT but utilizes noninvasive CD8 immunoPET imaging to stratify potential for secondary therapy [17, 38]. Further analysis of CD8-SUV in RT and RT → IO treated tumors identifies that CD8 immunoPET imaging stratifies response to combination RT → IO, rather than RT alone (Fig. 4E). CD8 immunoPET SUV serves as a short-term prognostic biomarker, providing an early post-radiotherapy metric associated with subsequent sensitivity to immunotherapy. While radiation is known to increase anti-tumoral immune activity, in our study we show that immune infiltration following RT is heterogeneous, allowing us to identify which tumors will be most receptive to IO.

This study has several important limitations related to model selection and experimental design that should be considered during interpretation of results. The lack of response to aPD1 + aCTLA4 in the radiosensitive parental 4T1 model is consistent with its immunologically ‘cold’ TIME and previously well-documented [33, 40, 54–58], which limits the efficacy of immune checkpoint blockade monotherapy. This study was designed to investigate the potential of radiation therapy to overcome this limitation by modulating the TIME and enhancing immune infiltration. The immunotherapy-only group was included as a baseline control to confirm the immunotherapy resistance of the 4T1 model (as previously demonstrated by our group and others [40]) and to contextualize the magnitude of response observed following radiation-primed immunotherapy. The primary analytical comparison in this study was between RT and RT followed by immunotherapy, rather than a direct comparison of RT followed by immunotherapy with immunotherapy alone (as immunotherapy provides limited to no benefit in this 4T1 model [59–61]). Although CD8 SUVmean can be modeled as a continuous variable, linear regression analysis demonstrated a weak and non-significant association with therapeutic response in this cohort, indicating that continuous modeling was less predictive than K_means_ stratification of high vs low expression, supporting the use of threshold-based analysis to capture differences in IO response. While results were promising, additional tumor models and doses of RT could expand personalized treatment strategies [62, 63], as others have shown there can be a “sweet spot” for radiation dose to synergize with immunotherapy [64]. Furthermore, differences in immunotherapy dosing schedules between immunotherapy alone and RT followed by immunotherapy reflect their distinct experimental purposes. Importantly, prior studies have demonstrated that the 4T1 model remains largely resistant to immunotherapy across a range of dosing schedules and treatment intensities, supporting that the limited response observed here is not attributable to dosing alone but reflects intrinsic resistance of this model [59–61]. Sustained immunotherapy exposure was therefore used to establish baseline response, while delayed immunotherapy administration was employed to specifically test radiation-induced immunotherapy priming. To our knowledge, this is the first study to utilize CD8 immunoPET imaging to monitor RT response and stratify responders to IO in a TNBC radiation sensitive and radiation resistant model.

Strategies involving CD8 immunoPET imaging have potential for clinical translation into cancer subtypes that are treated with standard-of-care RT, such as HNSCC or glioblastoma [37, 65–67]. Quantifying the dynamics of CD8 during RT may be used to inform timing and guidance of RT + IO, thereby maximizing therapeutic ratio on a precise and personalized level. In this framework, CD8-targeted immunoPET imaging could be performed shortly after RT to identify tumors exhibiting favorable immune modulation, thereby informing patient selection and sequencing for combination radiation-IO without requiring repeated invasive biopsies. Tumors with high CD8 immunoPET SUV following RT may exhibit better tumor control due to an intrinsic immune response. However, our findings indicate that the stratification provided by CD8 immunoPET SUV is particularly valuable in the context of IO, as high SUV tumors showed markedly improved responses to IO compared to low SUV tumors. This suggests that CD8 immunoPET imaging can serve as a powerful tool to identify tumors that are primed for IO following RT, facilitating personalized treatment strategies. Because IO is associated with a range of immune-related adverse side effects, stratifying patients based on likelihood of therapeutic response is critical to minimize overtreatment of patients who may experience toxicity without clinical benefit [61]. As CD8 immunoPET imaging captures therapy-induced tumor immune modulation, our results support the broader use of CD8-targeted PET imaging, such as with [^89^Zr]Zr-Df-IAB42, to monitor immune responses elicited by other immunomodulatory therapeutics such as radionuclide therapy and chemotherapy [68–70].

## Supplementary Information

Below is the link to the electronic supplementary material.

Supplementary Material 1

Supplementary Material 2

Supplementary Material 3

Supplementary Material 4

Supplementary Material 5

Supplementary Material 6

Supplementary Material 7

Supplementary Material 8

Supplementary Material 9

Supplementary Material 10

## Data Availability

Datasets and materials are available upon reasonable request.
