# Supplementary figures and images for: CD8 immunoPET imaging to stratify response and guide combination immunotherapy and radiation in triple negative breast cancer
Source: Breast Cancer Res. 2026 Apr 25;28:107. doi: 10.1186/s13058-026-02286-9 (PMC13267631; doi:10.1186/s13058-026-02286-9)

Bad 51

7/1/23

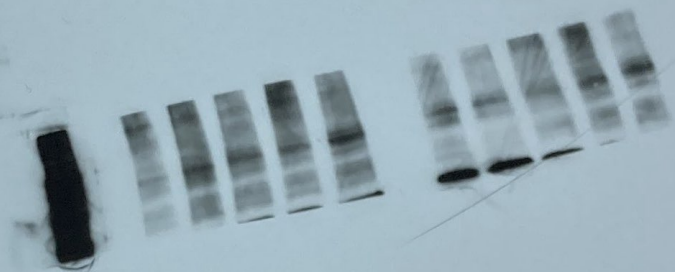

4T1

RR4T1

$\beta$ -actin

DNA-PKCS

Rad/51

7/1/23

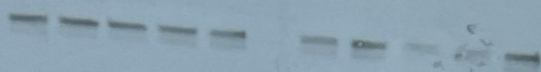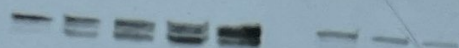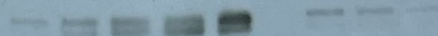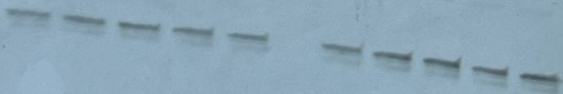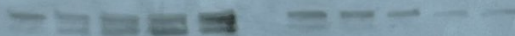

Supplement: Supplementary file 1 — Supplementary Material 1 [file 13058_2026_2286_MOESM1_ESM.pdf]

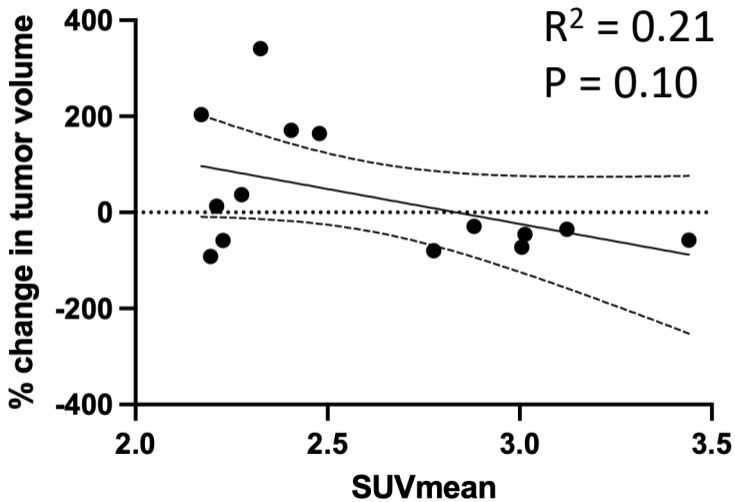

Supplement: Supplementary file 4 — Supplementary Material 4 [file 13058_2026_2286_MOESM4_ESM.pdf]

**A. Low CD8**

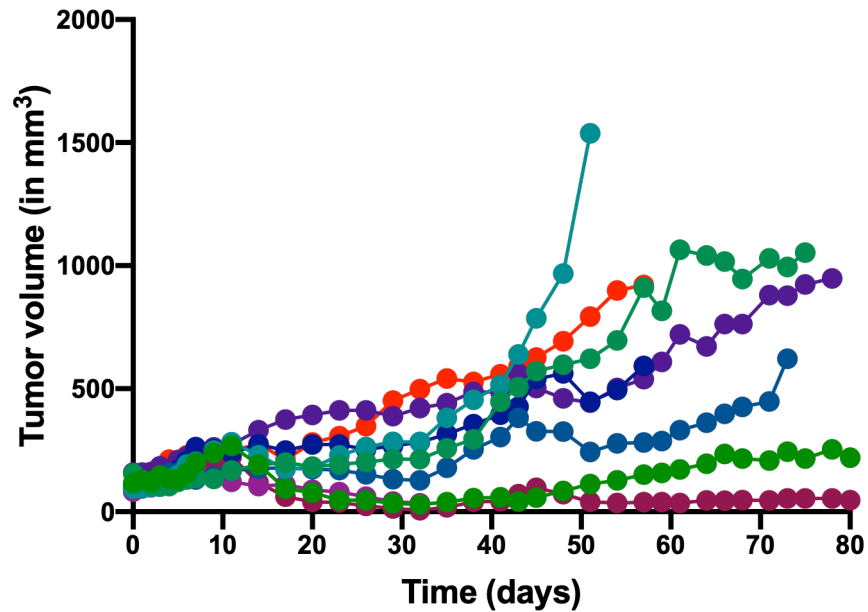

**B. High CD8**

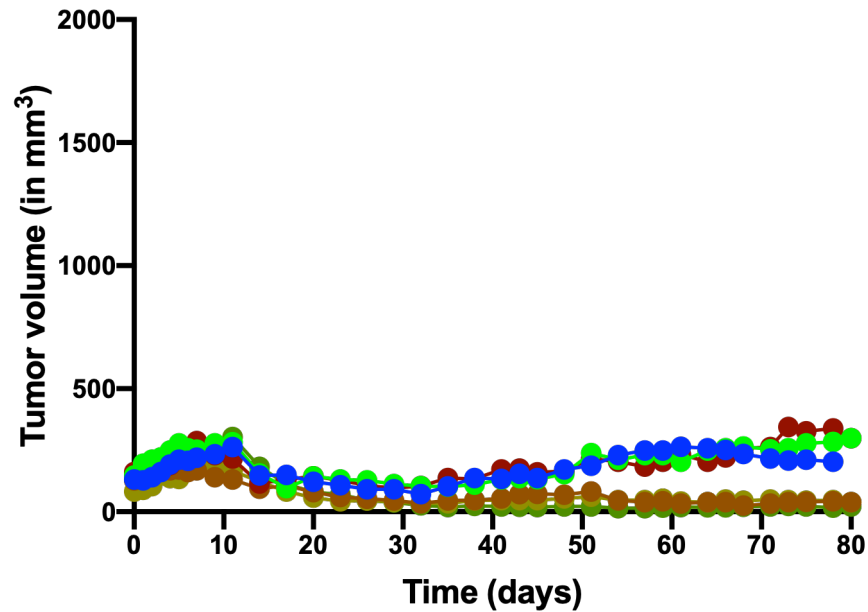

Supplement: Supplementary file 5 — Supplementary Material 5 [file 13058_2026_2286_MOESM5_ESM.pdf]

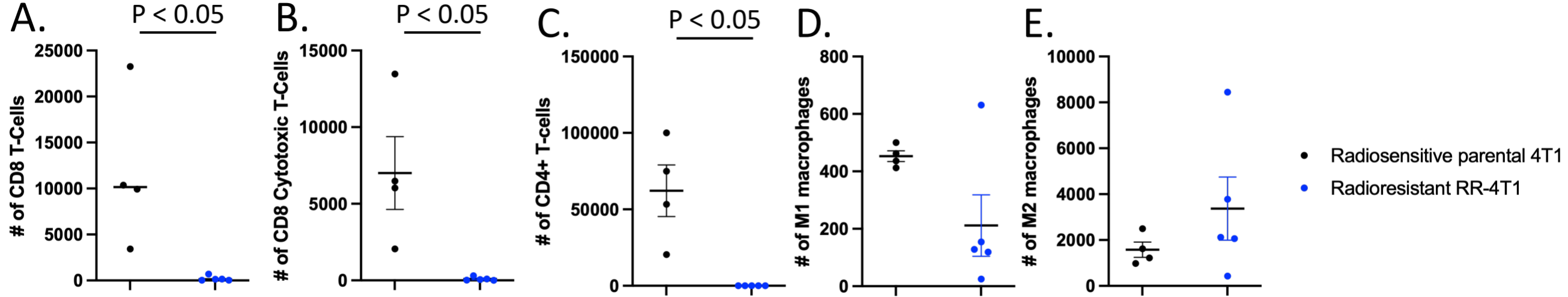

Supplement: Supplementary file 6 — Supplementary Material 6 [file 13058_2026_2286_MOESM6_ESM.pdf]

EMT6

Tumor SUV

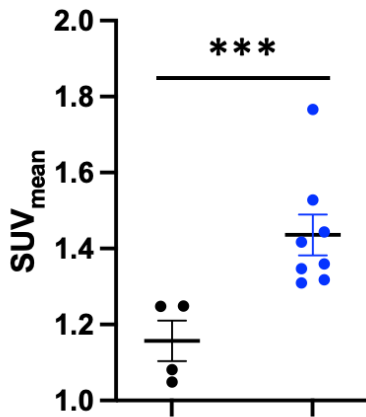

Muscle SUV

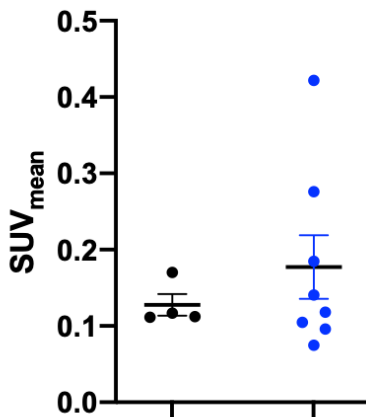

Heart SUV

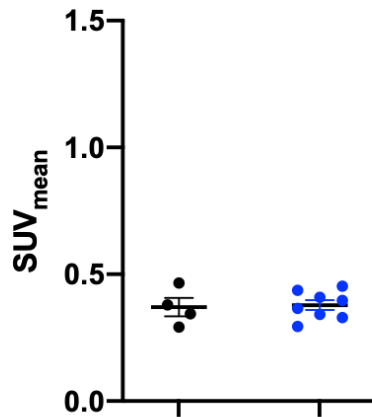

● Control  
● Radiation

Supplement: Supplementary file 7 — Supplementary Material 7 [file 13058_2026_2286_MOESM7_ESM.pdf]

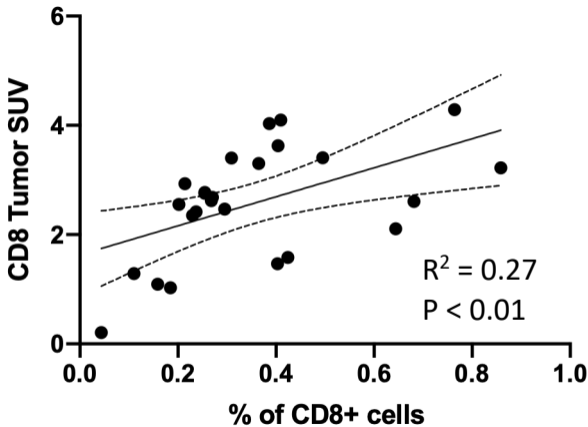

Supplement: Supplementary file 9 — Supplementary Material 9 [file 13058_2026_2286_MOESM9_ESM.pdf]

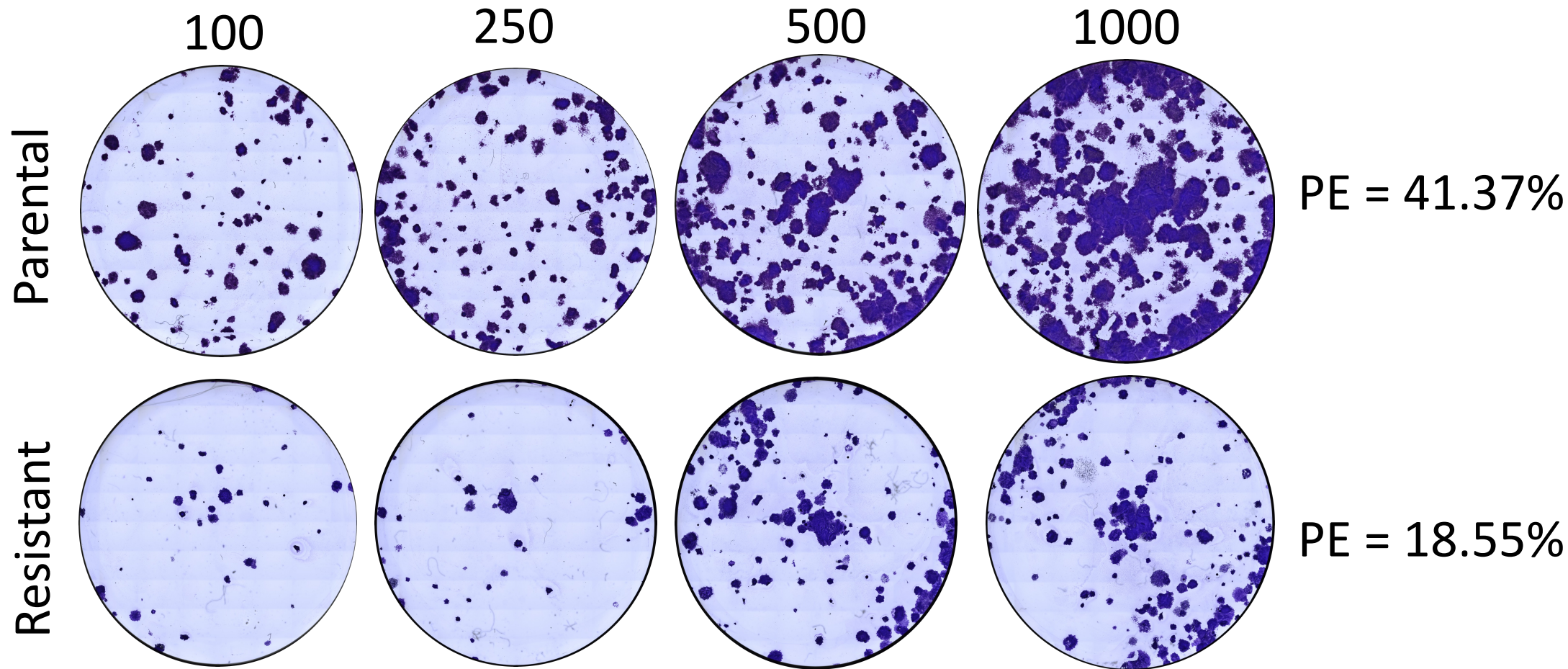

Supplement: Supplementary file 10 — Supplementary Material 10 [file 13058_2026_2286_MOESM10_ESM.pdf]
