# Supplementary material for: CD8 immunoPET imaging to stratify response and guide combination immunotherapy and radiation in triple negative breast cancer
Source: Breast Cancer Res. 2026 Apr 25;28:107. doi: 10.1186/s13058-026-02286-9 (PMC13267631; doi:10.1186/s13058-026-02286-9)
Supplement: Supplementary file 2 — Supplementary Material 2 [file 13058_2026_2286_MOESM2_ESM.docx]

% MATLAB script for γH2AX quantification
%
% Authors: Carlos A. Gallegos and Chloe T. DeMellier

% Lab: Anna Sorace, University of Alabama at Birmingham
% Date of most recent version: September 23, 2022

%

%%%%%%%%%%%%%%%%%%%%%%%%%%%%%%%%%%%%%%%%%%%%%%%%%%%%%%%%%%%%%

% Code for H2AX IHC staining quantification

function [percent] = TotDNADamage(DAPIimage, CY5image)

DAPIgray = im2gray(DAPIimage);

DAPIgray = imadjustn(DAPIgray);

DAPIthresh = graythresh(DAPIgray); % did use graythresh

bwDAPI = im2bw(DAPIgray,DAPIthresh);

a = ~bwDAPI;

b = -bwdist(a);

b = imhmin(b, 1);

b(a) = -Inf;

m = watershed(b);

im = bwDAPI;

im(m==0) = false;

bwDAPI = im;

bwDAPI = bwareaopen(bwDAPI, 100);

hblobDAPI = vision.BlobAnalysis( ...

'AreaOutputPort', true, ...

'BoundingBoxOutputPort', true, ...

'CentroidOutputPort', true, ...

'MinimumBlobArea',100, ...

'MaximumCount', 1000);

figure, imshow(bwDAPI)

[blobAreaDAPI, blobCentroidDAPI, ~] = hblobDAPI(bwDAPI);

numDAPI = length(blobAreaDAPI)

resultImage = bwDAPI;

resultImage = double(resultImage);

resultImage = insertMarker(resultImage, blobCentroidDAPI, 'x-mark', 'Color', 'red', 'Size', 20);

%figure, imshow(resultImage); title('DAPI count result');

CY5_signal = im2gray(CY5image);

regions=regionprops(bwDAPI,'Centroid','BoundingBox',"Image");

bb= {regions.BoundingBox};

c_shape= {regions.Image};

centroid = {regions.Centroid};

thresh1 = 120; % intensity threshold for γH2AX positivity (empirically determined and applied uniformly across all images)pad=1;

figure

imshow(CY5image)

hold on

for num= 1: length(bb)

% Get Data for each element

coor= bb{num};

image = c_shape{num};

% Generate coordinates for each cell

x= ceil(coor(1));

y= ceil(coor(2));

w= coor(3);

h= coor(4);

edges= [x-pad,x+w+pad*2-1,y-pad,y+pad*2-1];

% Isolate sections for each channel

CY5_sig=CY5image(y:y+h-1,x:x+w-1);

cell_signal = uint8(CY5_sig) .* uint8(image);

average_cell_signal(num) = mean(cell_signal(cell_signal>0), 'all');

center = centroid{num};

x = round(center(1));

y = round(center(2));

text(x,y, num2str(num), 'Color', 'blue', 'FontSize', 10);

end

numCY5 = [average_cell_signal >= thresh1];

numCY5 = numel(numCY5(numCY5 == 1));

percent = (numCY5/numDAPI)*100

end
