# Supplementary material for: CD8 immunoPET imaging to stratify response and guide combination immunotherapy and radiation in triple negative breast cancer
Source: Breast Cancer Res. 2026 Apr 25;28:107. doi: 10.1186/s13058-026-02286-9 (PMC13267631; doi:10.1186/s13058-026-02286-9)
Supplement: Supplementary file 3 — Supplementary Material 3 [file 13058_2026_2286_MOESM3_ESM.pdf]

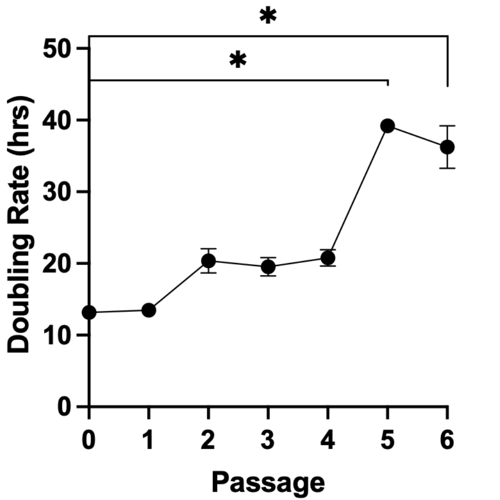

*
*
50
40
30
20
10
0
Doubling Rate (hrs)
0
1
2
3
4
5
6
Passage
