# Supplementary material for: CD8 immunoPET imaging to stratify response and guide combination immunotherapy and radiation in triple negative breast cancer
Source: Breast Cancer Res. 2026 Apr 25;28:107. doi: 10.1186/s13058-026-02286-9 (PMC13267631; doi:10.1186/s13058-026-02286-9)
Supplement: Supplementary file 8 — Supplementary Material 8 [file 13058_2026_2286_MOESM8_ESM.pdf]

| Cell line | $\alpha$ ($Gy^{-1}$) | $\beta$ ($Gy^{-2}$) | $\alpha/\beta$ (Gy) |
|---|---|---|---|
| Parental radiosensitive 4T1 | 0.17 ± 0.03 | 0.02 ± 0.01 | 9.3 |
| Radioresistant 4T1 | 0.01 ± 0.01 | 0.01 ± 0.01 | <1 |
